# Insect herbivory on seedlings of rainforest trees: Effects of density and distance of conspecific and heterospecific neighbors

**DOI:** 10.1002/ece3.4698

**Published:** 2018-12-07

**Authors:** Harriet Downey, Owen T. Lewis, Michael B. Bonsall, D. Catalina Fernandez, Sofia Gripenberg

**Affiliations:** ^1^ Department of Zoology University of Oxford Oxford UK; ^2^ Department of Biological Sciences University of Windsor Windsor Ontario Canada

**Keywords:** apparent competition, density‐dependence, Janzen–Connell hypothesis, natural enemies, seedling survival, tropical forest

## Abstract

Natural enemies of plants such as insect herbivores can contribute to structuring and maintaining plant diversity in tropical forests. Most research in this area has focused on the role of specialized enemies and the extent to which herbivory on individual plant species is density‐dependent. Relatively few insect herbivores specialize on a single host plant species. Insect herbivores that feed on more than one plant species may link the regeneration dynamics of their host species through “apparent competition” or “apparent mutualism.” We investigated herbivory and survival of seedlings of two tropical tree species (*Cordia alliodora* and *Cordia bicolor*) in the forests of Barro Colorado Island (Panama). We used experiments and observations to assess seedling fate in relation to the presence of conspecifics and heterospecifics across a range of spatial scales. Herbivory significantly increased seedling mortality and was highest at high local densities of *C. alliodora *seedlings. There was also evidence that high local densities of *C. alliodora* increased herbivory on co‐occurring *C. bicolor *seedlings. *Synthesis. *The elevated rates of seedling herbivory at high densities of conspecifics documented in our study are consistent with the predictions of the Janzen–Connell hypothesis, which explains how so many plant species can coexist in tropical forests. Our data also highlight the possibility that herbivore‐mediated density‐dependence, facilitated by herbivores that feed on multiple plant species, can also occur *across* plant species. Enemy‐mediated indirect effects of this sort have the potential to structure plant communities.

## Resumen

Los enemigos naturales de plantas, tales como insectos herbívoros pueden contribuir a estructurar y mantener la diversidad arbórea en los bosques tropicales. La mayoría de investigaciones en ésta área se han centrado en el papel de enemigos especializados y en la medida en la que la herbivoría en plantas hospederas individuales depende de la densidad.Relativamente pocos insectos herbívoros se especializan en una única especie de planta hospedera. Los insectos herbívoros que se alimentan en más de una especie de planta pueden vincular la dinámica de regeneración de sus hospederas a través de “competencia aparente” o “mutualismo aparente”.Nosotros investigamos la herbivoría y supervivencia de las plántulas de dos especies de árboles tropicales (*Cordia alliodora* y *Cordia bicolor*) en los bosques de la Isla de Barro Colorado (Panamá). Usamos experimentos y observaciones para evaluar el destino de las plántulas con relación a la densidad de conespecíficos y heteroespecíficos a través de un rango de escalas espaciales.La herbivoría aumentó significativamente la mortalidad de las plántulas, y fue significativamente más alta en densidades locales altas de *C. alliodora*. Hubo también evidencia que densidades locales altas de *C. alliodora* aumentaron la herbivoría en plántulas adyacentes de *C. bicolor*.*Síntesis.* Las elevadas tasas de herbivoría en plántulas a densidades altas de conespecíficos documentadas en nuestro estudio son consistentes con las predicciones de la hipótesis de Janzen‐Connell, la cual provee una explicación a la alta diversidad de especies vegetales en los bosques tropicales. Nuestros datos también resaltan la posibilidad que efectos similares mediados por herbivoría puedan ocurrir *a través de *especies y sean facilitados por herbívoros especializados al nivel de género en lugar de especies. Efectos indirectos de ésta clase mediados por enemigos tienen el potencial de estructurar comunidades vegetales.

## 1 INTRODUCTION

Plants and their insect herbivores account for around half of the macroscopic diversity on earth. The response of herbivores to the distribution of their host plants in the landscape can have profound consequences for plant population dynamics and community structure (Crawley, 1989; Maron & Crone, 2006).

Insect herbivores are thought to play a key role in plant regeneration dynamics, particularly in highly diverse tropical forest tree communities (Connell, 1971; Janzen, 1970). The Janzen–Connell hypothesis predicts that host‐specific pests such as insect herbivores will cause seed and seedling mortality to be highest in areas where the density of conspecifics is high, and/or in the vicinity of conspecific adults. This mechanism can facilitate high local plant diversity in tropical forests by limiting population growth of species that are common and favoring locally rare species (Connell, 1971; Janzen, 1970).

The Janzen–Connell hypothesis relies on enemies being reasonably host specific (Sedio & Ostling, 2013). In terms of their diet breadths, insect herbivores are known to range from specialists to generalists that attack a wide range of species (Novotny et al., 2010). In these instances, host species sharing one or more enemies could experience enemy‐mediated interspecific density‐dependence, increasing or decreasing each other's mortality rates via mechanisms such as “apparent competition” and “apparent mutualism” in locations where they co‐occur (Abrams, Holt, & Roth, 1998; Holt, 1977). This could lead to the regeneration dynamics of plant species being linked to heterospecific as well as conspecific plant densities, and to non‐specialist enemies shaping distributions of plant species at the community level.

The importance of enemy‐mediated indirect interactions in ecological communities has been demonstrated in many systems (e.g., Morris, Lewis, & Godfray, 2004; Muller & Godfray, 1999; Tompkins, Draycott, Hudson, 2000). Despite this, the role of these types of interactions and their relative importance compared to effects produced by specialist enemies have received little attention in tropical plant communities (but see Garzon‐Lopez et al.., 2015).

Numerous studies in tropical forests have investigated patterns of herbivory and/or plant survival in relation to either conspecific plant densities (reviewed in Comita et al., 2014) or the composition of the surrounding plant community (Weiblen, Webb, Novotny, Basset, & Miller, 2006). In most cases, the identity and diet breadth of the herbivores remains unknown, making it difficult to assess the likely mechanisms behind observed patterns. Moreover, the extent to which observed patterns of herbivory suppress plant performance is typically unknown (but see Bagchi et al., 2014), making it challenging to infer the likely ecological impact of the herbivores.

We used experiments and field surveys to investigate patterns of herbivory on seedlings of two closely related tropical tree species, *Cordia alliodora *(Ruiz & Pav. Oken) and *Cordia bicolor *(A.D.C.) (Boraganaceae). During preliminary studies (field observations and feeding trials in the laboratory), the tortoise beetle *Ischnocodia annulus* (Fabricus 1781) (Coleoptera: Chrysomelidae) was identified as an abundant insect herbivore shared by *C. alliodora* and *C. bicolor.*

We investigated patterns of herbivory in relation to host plant density and assessed the potential for recruitment dynamics of the two *Cordia* species to be coupled via the foraging behavior of the shared enemy. More specifically, our studies were designed to address the following questions: (1a) Do *Cordia* seedlings experience intra‐ and/or interspecific density‐dependent seedling herbivory; and if so, (1b) at what spatial scale do any density effects occur? (2a) Is herbivory of *Cordia *seedlings higher closer to conspecific and congeneric adults than elsewhere in the landscape; and (2b) if so, at what distance do herbivory levels decline? (3) Does herbivory affect *Cordia* seedling survival?

Our results exploring the effects of conspecific distance‐ and density‐dependent herbivory are relevant in the context of the Janzen–Connell hypothesis. Measuring the effects of congeneric distance‐ and density‐dependent herbivory allows us to assess whether enemy‐mediated indirect interactions between *Cordia* species occur at our study site.

## 2 MATERIALS AND METHODS

### 2.1 Study site

Barro Colorado Island (BCI) is a 15.6 km^2^ island located in Gatun Lake, Panama, (Lat: 9.1543, Long: −79.846) that was isolated from the surrounding mainland in 1914 when the Rio Chagres was dammed to form part of the Panama Canal. The island is covered with tropical semi‐deciduous forest. The northeast half experienced widespread clearing during the late 1800s, whilst the other half has been little disturbed for up to 1,500 years. BCI's climate is seasonal, with a dry season from December to April/May and an average yearly rainfall of 2,612 mm (Leigh, 1999).

Most investigations presented in this study were carried out in four mapped forest plots (one ten hectare, one twenty‐five hectare and two six hectare; Supporting Information Figure S1) that were established in the mid‐1990s. In these plots, every free‐standing woody stem larger than 20 cm diameter at breast height (dbh) has been tagged, measured (dbh), and identified to species level. These plots were last censused in 2013 and 2014.

### 2.2 Study system

The genus *Cordia *(Boraginaceae) consists of trees, shrubs, lianas, vines, and erect herbs. Three species occur on BCI and overlap in their reproductive phenology, with the majority of seeds germinating in May and June. *Cordia alliodora* is a tall pioneer tree species with wind‐dispersed seeds that germinate to form dense seedling carpets in the vicinity of reproducing trees. *Cordia bicolor* is typically slightly smaller than *C. alliodora* and produces single‐seeded fruits (ca 1.5 cm in length) that are dispersed by mammals and birds. *Cordia lasiocalyx* is a small tree with fruit that resembles those of *C. bicolor *(Croat, 1978)*. *Since its dbh rarely exceeds 20 cm, it does not appear in the plot data sets, and seeds and seedlings of *C. lasiocalyx* appear to be considerably less common on BCI than those of *C. alliodora* and *C. bicolor*. For these reasons, we omitted *C. lasiocalyx* from our studies.

**Figure 1:**
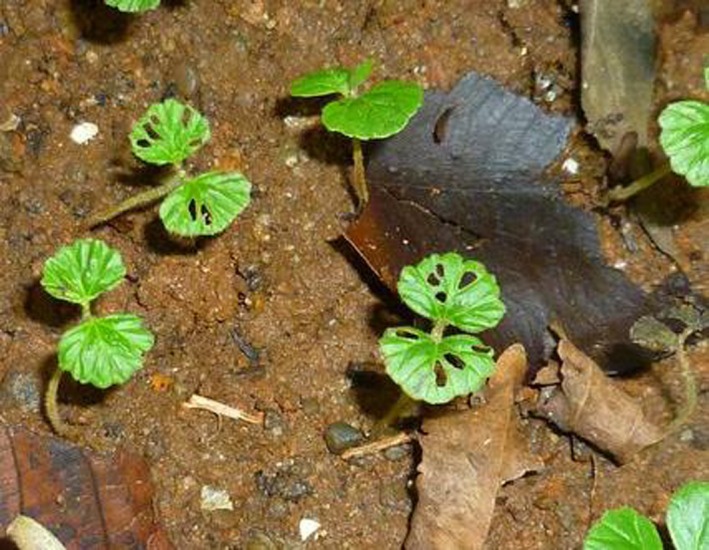
Feeding marks of *Ischnocodia annulus *on newly germinated *Cordia alliodora* seedlings

At our field site, newly germinated seedlings of all three species are attacked by the target beetle *I. annulus* (Chrysomelidae) (Figure 1). Data from a long‐term Malaise trap study on BCI show that the activity of *I. annulus* peaks during May and June, when *Cordia* seedlings germinate (D.M. Windsor, personal communication). Whilst we are confident that *I. annulus* causes the majority if not all of the herbivory to young *Cordia* seedlings, we cannot completely exclude the possibility that other insect herbivores also feed on *Cordia* seedlings. As our study is focused on enemy‐mediated density‐dependence and enemy‐mediated indirect interactions, this will not change the interpretation of our results.

Whilst pathogens have been shown to cause substantial mortality in young seedlings of other tropical tree species (e.g., Augspurger, 1984; Bell, Freckleton, & Lewis, 2006), the number of seedling with signs of pathogen attack was negligible in our studies (only 0.02% of 4,120 individuals showing signs of pathogen attack throughout our studies). We are therefore confident that insect herbivores are the most important enemies of young *Cordia* seedlings in our study.

### 2.3 Data collection

To address our study questions, we used a combination of experimental work and observational surveys on natural *Cordia* seedlings conducted across three field seasons (2013, 2015, and 2016).

For experiments, newly germinated seedlings were collected beneath reproducing *C. alliodora* trees near the laboratory clearing in the northeastern part of BCI. Seedlings were placed individually in pots filled with sterilized soil and allowed to acclimatize in a shade house for a minimum of two days before being taken to the field (still in their pots). All seedlings were taken to the field at the cotyledon stage. Experimental seedlings were free from insect herbivores and showed no evidence of pathogen attack when experiments were initiated.

Specific study methods to address each question are described below. Table 1 provides an overview of the questions addressed by each study.

**Table 1:** Summary of study questions addressed by each study

Study	Do *Cordia* seedlings experience density‐dependent seedling herbivory?	At what spatial scale do conspecific and congeneric density effects occur?	Is herbivory of seedlings higher close to conspecific and congeneric adults than elsewhere in the landscape?	At what distance from adult trees do seedling herbivory levels decline?	Is *Cordia* seedling survival lower on seedlings with herbivory than on intact seedlings?
1	✓	✓			
2	✓				
3	✓		✓	✓	
4			✓	✓	✓
5					✓

#### 2.3.1 Do *Cordia* seedlings experience density‐dependent seedling herbivory; and if so, at what spatial scale do conspecific and congeneric density effects occur?

##### Study 1

To investigate how *C. alliodora* seedling herbivory was affected by the density of conspecific seedlings at a small spatial scale, and by the distribution of adults of *C. alliodora *and *C. bicolor* at a large spatial scale, experimental seedlings were introduced to the 25 ha mapped plot (Supporting Information Figure S1). The 25 ha mapped plot was divided into 625 20 × 20 m subplots (Supporting Information Figure S2) using an existing grid of PVC markers. To minimize problems with edge effects, we excluded all subplots within 40 m of the mapped plot edge. In each of the remaining 441 subplots, seedlings were placed at nine locations, guided by the existing PVC markers: one in each of the corners, one halfway along each edge of the subplot, and one in the center (Supporting Information Figure S2). At one of these nine locations, selected at random, we placed a cluster of eight potted seedlings. A single potted seedling was placed at the remaining eight locations. Thus, there were 16 seedlings in total per subplot, eight in a “high” density and eight in a “low” density treatment.

The experiment was established between 17th May and 3rd June 2016 with seedlings placed in 120 subplots. In total, 1,869 seedlings were placed at 1,089 locations (PVC poles). It was not possible to place seedlings at all nine locations in 20 subplots because of steep terrain, lianas or dense undergrowth. All introduced seedlings were monitored weekly for the first month, then every 10 days. In total, we completed seven censuses, with the last census taking place on 5th August 2016. At each census, seedlings were scored for presence or absence of herbivory. To quantify the abundance of adult *Cordia* trees in the study area, every mapped adult of *C. alliodora* and *C. bicolor* in the 25 hectare plot (including those close to plot edges) was visited and its reproductive status recorded during 15th and 31st May 2016. Adults were scored as reproductive if flowers, fruits, or recently established seedlings were observed.

##### Study 2

To assess levels of seedling herbivory in relation to conspecific and congeneric abundances, 27 20 × 20 m subplots located in three of the mapped forest plots (Supporting Information Figure S1) were surveyed. The 27 survey plots were selected to represent varying densities of *Cordia* adult individuals: no *Cordia*, one *C. alliodora*, one *C. bicolor*, multiple *C. alliodora*, multiple *C. bicolor*, low density (2–3 individuals) mixed species, and high density (>4 individuals) mixed species. Within each of these subplots, where access was possible, nine 0.5 m^2^ quadrats were set up, one at each corner of the subplot, one at the mid‐point of each subplot edge, and one in the center. In each quadrat, every newly germinated (i.e., current year) *C. alliodora* and *C. bicolor* seedling was counted and scored for presence or absence of herbivory. A total of 255 quadrats were surveyed, 105 of which had *Cordia *seedlings in them. Censuses took place from 25 May 2015 to 21 June 2015.

#### 2.3.2 Is herbivory of seedlings higher closer to conspecific and congeneric adults than elsewhere in the landscape; and if so, at what distance do herbivory levels decline?

##### Study 3

To assess whether seedlings of *C. alliodora* suffer elevated insect attack rates close to conspecifics and/or congeneric adult trees, and to explore the spatial extent of such effects, we introduced potted seedlings to 18 sites in the mapped forest plots and subsequently scored them for herbivory.

Six adults of each of *C. alliodora*, *C. bicolor, *and *Guapira standleyana *(Woodson) were selected from the four mapped plots. *Guapira standleyana *(Nyctaginaceae) was selected as a heterofamilial species to provide a non‐*Cordia* comparison. This species is similar in size and abundance to the studied *Cordia* species, but is not closely related to them and is not a host of *I. annulus*. The 18 sites were chosen as the adult trees were reproductive (i.e., seeds and/or seedlings present in their vicinity) and were separated from the nearest *Cordia* adult by a distance of at least 30 m. At each tree, a linear transect of 26 potted *C. alliodora *seedlings (hereafter referred to as “focal” seedlings) was established, oriented in a random direction, with single plants spaced at 1 m intervals from 0 m to 25 m. This distance was chosen to reflect the typical extent of natural *C. alliodora* seedling carpets observed in preliminary surveys (mean 18 m, maximum 23 m). If there was another *C. alliodora* tree within 30 m of a focal tree, we selected an alternative transect direction. All natural seedlings in a 0.5 m^2^ quadrat surrounding each focal seedling were counted. Focal seedlings were scored for presence or absence of herbivory every two or three days from 24 June 2015 until 15 July 2015, when herbivory had leveled off. This allowed us to obtain an accurate herbivory score for each focal seedling, even in cases where a focal seedling later died.

##### Study 4

To assess whether seedlings of *C. alliodora* and *C. bicolor* experience elevated herbivory close to reproducing *C. alliodora* adults, potted *C. alliodora and C. bicolor* seedlings were introduced (still in their pots) to sites close to each of four *C. alliodora* trees. Seedlings of both species were obtained from seeds collected on BCI and germinated in a shade house. At each tree, 11 *C. alliodora* and 11 *C. bicolor* seedlings were positioned randomly within an area of approximately 2 × 2 m within the seedling carpet of the adult tree. The precise location was selected haphazardly from sites with suitable terrain. An additional 11 *C. alliodora* and 11 *C. bicolor* seedlings were placed within a similar‐sized area further away from each adult tree, outside the seedling carpet, but within five meters of the periphery of the seedling carpet. We refer to seedlings in the two distance categories as “near” and “far” seedlings, respectively. Seedlings were transferred to the field between 5th and 29th July 2013 and revisited to score herbivory (presence/absence) and survival (alive/dead) approximately every two days until 28th August 2013, when herbivory had leveled off.

#### 2.3.3 Does herbivory affect *Cordia* seedling survival?

Survival of *C. alliodora* and *C. bicolor* seedlings from Study 4 (above) following herbivory was recorded. To determine how herbivory co‐varies with *C. alliodora* seedling survival, two *C. alliodora* seedling carpets were selected on 1 June 2015. Within each seedling carpet, 20 intact seedlings and 20 seedlings with herbivory were haphazardly selected, numbered, and their fate (alive or dead) followed up for six weeks.

### 2.4 Statistical analysis

*Study 1: *Data were analyzed using Generalized Linear Models (GLMs) with quasibinomially distributed errors (to account for underdispersion). To test for spatial autocorrelation in herbivory, focal seedling herbivory (a binary response) was modeled as a function of the proportion of seedlings with herbivory within 10 different radii (from 0 to 100 m). Additionally, the proportion of seedlings with herbivory within a subplot was modeled as a function of seedling treatment (high or low density), summed basal area of *C. alliodora *adults, and summed basal area of *C. bicolor *adults within the neighborhood. Subplot was used as a random effect. After trying several different neighborhood distances, 50 m from the central point of each subplot was selected as the most appropriate by comparing model AIC values and was thus used in the final model.

*Study 2*: Numbers of *C. bicolor* seedlings in our observational plots were too low to test for intra‐ or interspecific density effects on *C. bicolor* herbivory rates. Data on *C. alliodora* seedling herbivory were analyzed using a Generalized Linear Mixed Effects Model (GLMM) with binomially distributed errors. Seedling herbivory (proportion of seedlings with signs of herbivory per 0.5 m^2^ quadrat) was modeled as a function of local density of *C. alliodora* seedlings (in 0.5 m^2^ subplot), local density of *C. bicolor* seedlings (in 0.5 m^2^ subplot), density of *C. alliodora* adults in plot (20 m × 20 m), density of *C. bicolor* adults in plot (20 m × 20 m), summed basal area of *C. alliodora* adults in plot (20 m × 20 m), and summed basal area of *C. bicolor *adults in plot (20 m × 20 m) (all fixed effects). Plot number was included as a random effect to account for non‐independence of seedlings within the same plot. To account for overdispersion in the data, an observation‐level random factor was included in the model. Here, each data point receives a unique level of a random effect that models the extra variation present.

*Study 3: *Data were analyzed using GLMMs with binomially distributed errors. Seedling herbivory at the final census on each focal seedling (a binary response) was modeled as a function of the distance (m) of the seedling from adult tree, local seedling density (the number of natural *Cordia* seedlings in the 0.5 m^2^ quadrat surrounding the focal seedling), adult tree species (*C. alliodora*, *C. bicolor,* or *G. standleyana*), and the interaction between adult tree species and local seedling density (all fixed effects). Adult tree identity was included as a random effect to account for non‐independence of seedlings within the same transects.

*Study 4: *Data were analyzed using GLMs with quasibinomially distributed errors (to account for overdispersion). Seedling herbivory and seedling survival at the final census (binary response) were both modeled as a function of seedling location (inside/outside seedling carpet), focal seedling species (*C. alliodora* or *C. bicolor*), number of days the seedling was in the field, and the interaction between distance from adult tree and focal seedling species. Adult tree identity was included as a fixed effect rather than a random effect, as there were only four levels.

*Study 5:* Data were analyzed with a GLM with binomially distributed errors. Seedling survival (a binary response) was modeled as a function of initial seedling state (intact/herbivory).

In all analyses, the maximal models (including all fixed effects) were simplified by sequentially dropping terms until arriving at a minimum adequate model following the procedures recommended by Crawley (2007). Maximal and minimal adequate models for all analyses are shown in Supporting Information Table S1. All analyses were run in RStudio, R version 3.3.1 (2016‐06‐21)—“Bug in Your Hair” using the packages lme4 (Bates, Maechler, Bolker, & Walker, 2015) and multcomp (Hothorn, Bretz, & Westfall, 2008) for statistical analyses, and ggplot2 (Wickham, 2009), for figures.

## 3 RESULTS

### 3.1 Do *Cordia* seedlings experience density‐dependent seedling herbivory; and if so, at what spatial scale do conspecific and congeneric density effects occur?

**Figure 2:**
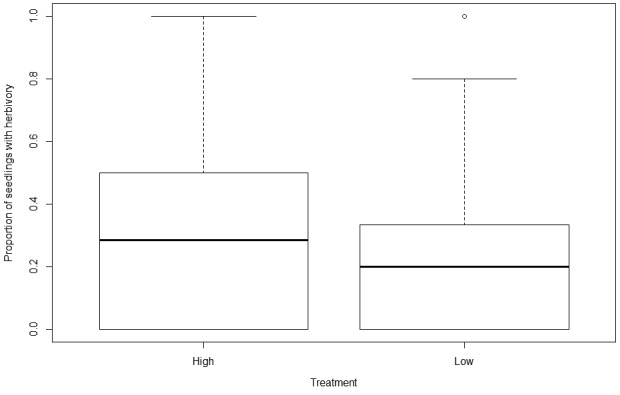
Proportion of seedlings with herbivory in high (eight seedling) and low (one seedling) density treatments

In Study 1, the proportion of *C. alliodora *seedlings experiencing herbivory was significantly lower in the low density (one seedling treatment) than in the high density (eight seedlings) (β = −0.4389, *SE* = 0.2071, *t* = −2.119, *p* = 0.0354; Figure 2). The likelihood of focal *Cordia* seedlings being attacked by herbivores was not affected significantly by the summed basal area of either conspecific or congeneric adults in the neighborhood (conspecifics: Deviance = −0.0203, *F* = 0.0516, *p* = 0.8205; congenerics: Deviance = 0.3177, *F* = 0.8125, *p* = 0.3685). Study 1 revealed no evidence of spatial autocorrelation in herbivory of *C. alliodora *seedlings across the twenty‐five hectare plot (see Supporting Information Table [Link], [Link]). Whilst some distances came out as significant in some analyses, this was not consistent over time periods (see Supporting Information Table [Link], [Link]).

In Study 2, a total of 788 *C. alliodora* seedlings were present in the surveyed plots, of which 388 (49.2%) had signs of herbivory. Only eight *C. bicolor* seedlings were observed, which is too few to analyse. There were no significant effects of basal area of either *C. alliodora* or *C. bicolor* adults on the likelihood of seedling herbivory (*C. alliodora*: χ^2^ = 1.5632, *df *= 1, *p* = 0.2112; *C. bicolor*: χ^2 ^= 0.0159, *df *= 1, *p* = 0.8996). In Study 2, there was a trend for *C. alliodora* seedlings surrounded by high numbers of conspecific seedlings in the immediate (0.5 m^2^) neighborhood to be more likely to experience herbivory (β = 0.034, *SE* = 0.018, *z* = 1.917, *p* = 0.055).

### 3.2 Is herbivory of seedlings higher closer to conspecific and congeneric adults than elsewhere in the landscape; and if so, at what distance do herbivory levels decline?

**Figure 3:**
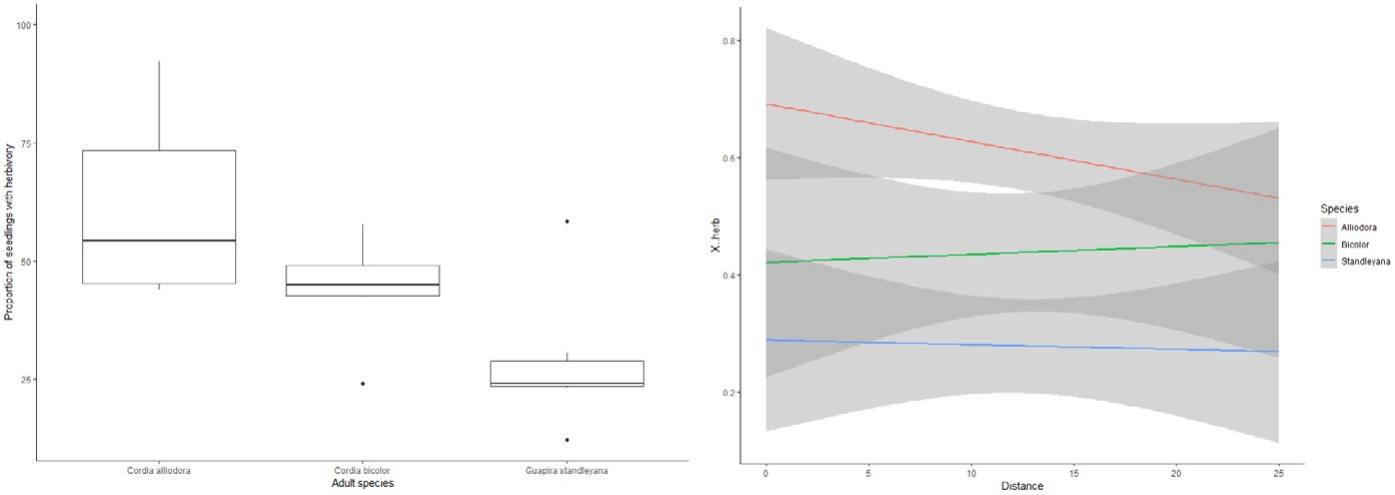
(Left) The percentage of *Cordia alliodora* seedlings placed close to conspecific, congeneric (*Cordia bicolor*), and heterofamilial (*Guapira standleyana*) attacked by *Ischnocodia annulus* during the course of Study 3. Whiskers represent lowest and highest values (outliers excepted), horizontal lines of the boxes refer to q25, median and q75 (bottom to top), and black dots represent outliers (Right) Proportion of seedlings with herbivory at distances (0–25 m) from conspecific, congeneric (*C. bicolor*), and heterofamilial (*G. standleyana*) trees, note all trends are non‐significant

In Study 3, of the 468 focal seedlings, 42% had experienced herbivory by the end of the experiment: 58%, 41%, and 27% under *C. alliodora, C. bicolor,* and *G. standleyana*, respectively. Neither the distance from an adult tree nor local seedling density significantly affected the likelihood of herbivory on focal *Cordia* seedlings (Distance: χ^2^ = 2.7822, *df *= 1, *p* = 0.09; Density: χ^2^ = 0.41, *df *= 1, *p* = 0.5243). The only fixed effect in the final model was adult tree species: the likelihood of potted *C. alliodora *seedlings being attacked differed significantly between adult tree species (χ^2^ = 10.37, *df *= 2, *p* = 0.006). More specifically, *C. alliodora* seedlings were less likely to experience herbivory close to heterofamilial adults (i.e., *G. standleyana*) than under conspecific adults (β = −1.474, *SE* = 0.404, *z* = −3.648, *p* < 0.001). There was also a trend for *C. alliodora *seedlings to experience less herbivory close to congeneric *C. bicolor *adults than under conspecific adults (β = −0.756, *SE* = 0.395, *z* = −1.915, *p* = 0.055) and for seedlings close to *G. standleyana* to experience less herbivory than those close to *C. bicolor *adults (β = −0.719, *SE* = 0.399, *z* = −1.800, *p* = 0.072) (Figure 3).

**Figure 4:**
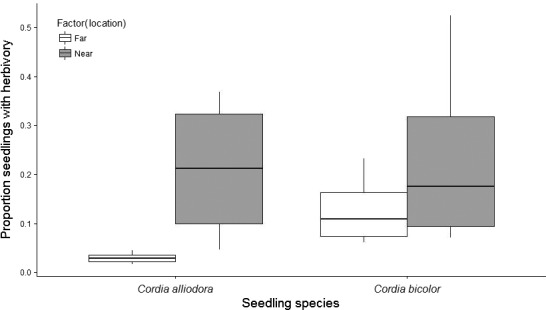
Proportion of seedlings with herbivory by *I. annulus *at different distances (near =inside seedling carpet, far=outside seedling carpet) from adult *Cordia alliodora* trees. Whiskers represent lowest and highest values, the horizontal lines of the boxes show q25, median and q75 (bottom to top), and black dots represent outliers

In Study 4, 59% of the 176 focal seedlings experienced herbivory (*C. alliodora* near 74%, *C. alliodora* far 32%, *C. bicolor* near 74%, *C. bicolor *far 56%). All terms were retained in the final model. The significant interaction between distance and species (χ^2^ = 39.234, *df *= 1, *p* < 0.001) indicates that the magnitude of the distance effects on seedling herbivory differs between the two species. Seedlings of both *C. alliodora *and *C. bicolor *placed close to *C. alliodora* adults experienced higher herbivory than those placed further away (Figure 4). This distance effect was significant for both species (*C. alliodora*: β = 1.891, *SE* = 0.126, z = 14.970, *p* < 0.001, *C. bicolor*: β = 0.797, *SE* = 0.123, z = 6.506, *p* < 0.001), but was more pronounced for *C. alliodora*. Seedlings of *C. bicolor* experienced significantly higher levels of herbivory than those of *C. alliodora *outside seedling carpets (β = 1.086, *SE* = 0.119, z = 9.144, *p* < 0.001; Figure 4) but not within seedling carpets (β = 0.008, *SE* = 0.129, *z* = −0.064, *p* = 1.000; Figure 4). The likelihood of being attacked increased significantly with time (deviance = 21.71, *df *= 1, *p* < 0.001) and also differed among sites (deviance = 51.024, *df *= 1, *p *= <0.001).

### 3.3 Does herbivory affect *Cordia* seedling survival?

In Study 5, seedlings of *C. alliodora *were significantly less likely to survive to the end of the experiment if they experienced herbivory (7% survival) than those that did not (40% survival, β = −2.107, *SE* = 0.682, *z* = −3.091, *p* = 0.002; Supporting Information Figure S3). Similarly, in Study 4, seedlings with herbivory were more likely to experience mortality than those without (β = 4.555, *SE* = 0.265, z = 17.170, *p* < 0.001). The proportions of seedlings that survived throughout the experiment with or without herbivory were 42% and 99%, respectively. The effect of herbivory on seedling survival did not differ significantly between species.

Outputs for all models can be seen in Supporting Information Table S3.

## 4 DISCUSSION

Our studies of patterns of herbivory on seedlings of two tropical tree species revealed that herbivory is common and widespread in young (<2 months) *Cordia* seedlings. Across data sets, 25%–59% of the surveyed seedlings showed signs of herbivory. Seedlings subject to herbivory were less likely to survive than unattacked seedlings, suggesting that patterns of herbivory may translate into patterns of seedling recruitment. The results from our analyses revealed intra‐specific density‐dependence in one species (*C. alliodora*) and the potential for enemy‐mediated interspecific interactions between the two studied *Cordia* species.

### 4.1 Do *Cordia* seedlings experience density‐dependent seedling herbivory?

Consistent with predictions of the Janzen–Connell hypothesis (Connell, 1971; Janzen, 1970) and results from numerous empirical studies (Comita et al., 2014), we found evidence for small‐scale density‐dependent herbivory in *C. alliodora *seedlings. In contrast with many other studies (but see Bagchi et al., 2014; Bell et al., 2006), we have also identified the likely mechanism driving these patterns: herbivory by insects, particularly the beetle *I. annulus*.

The documented small‐scale density‐dependence in herbivory suggests a behavioral response by herbivores, which may be more likely to locate clusters of seedlings than more isolated individuals and to feed on multiple plants in such a cluster. Aggregative responses of this sort by herbivores can stabilize consumer–resource interactions (Hassell & May, 1973, 1974 ; Hassell & Pacala, 1990). If responses of this sort are frequent in herbivore–plant interactions (e.g., Alvarez‐Loayza & Terborgh, 2011), this could favor plant species coexistence under the Janzen–Connell mechanism.

Although local seedling density (in the surrounding 0.5 m^2^) did not affect herbivory rates on focal seedlings in Study 4, herbivory was higher under conspecific adults (where there is a higher density of conspecific seedlings) than under congeneric or heterofamilial adults. This suggests that the density of conspecific seedlings does affect herbivory rates, but at a slightly larger scale than we tested.

### 4.2 At what spatial scale do conspecific and congeneric density effects occur?

We did not find evidence of density‐dependent herbivory at larger scales (20 × 20 m) in our studies. In both Study 1 and Study 2, herbivory did not increase with the summed basal area of adult trees (either *C. alliodora* or *C. bicolor*) in the wider neighborhood. These results are consistent with those of Zhu, Comita, Hubbell, and Ma (2015) who carried out a comprehensive study of density‐dependence for 50 North American tree species. They found that effects of conspecific density were pervasive, but the strength varied across life stages, with the impact of conspecific seedlings being stronger than that of conspecifics in other size classes. Similarly, a meta‐analysis of experimental studies of conspecific density‐dependence in seedlings found that Janzen–Connell effects were restricted to relatively small spatial scales (Comita et al., 2014). One possible explanation for the lack of density effects at larger spatial scales is that it is masked or counter‐acted by the heterogeneous effects of environmental suitability (Chen et al., 2010; Johnson, Condit, Hubbell, & Comita, 2017).

### 4.3 Is herbivory of seedlings higher closer to conspecific and congeneric adults than elsewhere in the landscape; and if so, at what distance do herbivory levels decline?

Previous studies of tropical trees suggest that distance‐responsive natural enemies can play a key role in seedling recruitment (Adler & Muller‐Landau, 2005; Swamy & Terborgh, 2010). Herbivory on both *Cordia* seedling species was significantly higher close to reproductive *C. alliodora *individuals than further away (outside seedling carpets) (Study 4). Since herbivory was associated with increased seedling mortality, this suggests that the recruitment of *C. alliodora *could be suppressed around adult trees, as envisaged by the Janzen–Connell hypothesis (Connell, 1971; Janzen, 1970). Furthermore, this mechanism could also lead to suppressed recruitment of *C. bicolor*, thus linking the regeneration dynamics of these two species.

Our transect experiment (Study 3) also revealed a trend for higher levels of herbivory close to adults of a congeneric species. Higher herbivory of *C. alliodora* seedlings close to adults of *C. alliodora* than close to adults of *C. bicolor* could result if the dense seedling carpets associated with *C. alliodora* (but not *C. bicolor*) are particularly attractive to enemies. Alternatively, herbivores may have a feeding preference for *C. alliodora *seedlings regardless of abundance, causing them to aggregate near the adult trees. Working in Peru, Alvarez‐Loayza and Terborgh (2011) found that mortality rates of conspecific seedlings within seedling carpets were much higher than those of heterospecific seedlings, but their study does not allow a comparison of the survival of these seedlings outside seedling carpets.

All transects of Study 3 (including heterofamilial *G. standleyana* transects) experienced herbivory, suggesting that *Cordia* enemies can readily disperse and locate seedlings over distances of 30 m (the minimum isolation distance from other *Cordia* adult trees). More extensive and systematic exposure of experimental seedlings, combined with widespread trapping of beetles would be needed to assess how enemy pressure varies across the landscape in relation to *Cordia* distances and densities.

### 4.4 Does herbivory affect Cordia seedling survival?

Herbivory was strongly associated with subsequent seedling mortality for both *Cordia* species. Whilst herbivory can be a symptom of plant stress (White, 1984), the direction of causality in this case is likely to be top‐down, with herbivores reducing plant fitness, perhaps by reducing the leaf surface area available for photosynthesis in the shaded forest understorey. Previous studies of tropical tree seedlings have documented similar results (Eichhorn, Nilus, Compton, Hartley, & Burslem, 2010; Norghauer & Newbery, 2014).

Herbivory thus appears to be an important source of mortality during the early months of seedling establishment. However, it is worth noting that, independent of herbivory, survivorship will inevitably be low for young seedlings of *C. alliodora,* which is a light‐demanding species with seedlings that are unable to persist for extended periods in the shaded understorey. Thus, if seedlings germinate and remain in shaded conditions, mortality will be inevitable within a few months or years unless a light gap opens nearby. Nonetheless, short‐term dynamics within seedling carpets could still determine the distribution of survivors in the longer term, providing the template on which later causes of morality act (Harms, Wright, Calderón, Hernández, & Herre, 2000). Further longer‐term study is needed to investigate the full implications of seedling mortality for *Cordia* population dynamics.

### 4.5 Community‐level consequences

In the shaded understorey of tropical forests, seedling densities are usually low, and competition between seedlings for light and other limiting resources is weak (Svenning, Fabbro, & Wright, 2008; Timothy Paine, Harms, Schnitzer, Carson&, 2008). This opens up the opportunity for alternative mechanisms, such as interactions with natural enemies, to play a role in maintaining and structuring plant diversity (Wright, 2002). In the case of *C. alliodora*, density‐dependent herbivory is likely to cause density‐dependent mortality. If other co‐occurring plant species show similar effects, this could promote species coexistence and diversity (Bagchi et al., 2010; Chen et al., 2010).

Our results also highlight potential for enemy‐mediated indirect interactions between *Cordia* species. We documented higher herbivory on *C. alliodora *seedlings close to adults of *C. bicolor *than close to non‐*Cordia* species and found elevated rates of herbivory of *C. bicolor *inside *C. alliodora *seedling carpets. Although our pattern‐based study does not reveal the longer‐term consequences of shared herbivory in the system, our observations suggest that enemy‐mediated indirect interactions with potential consequences for species recruitment might operate in this system. Positive aggregative responses by a consumer to multiple resource species can lead to short‐term apparent competition (Holt & Bonsall, 2017; Holt & Kotler, 1987): a negative relationship between the population growth rates of the resource species. Shared natural enemies can alter the balance of competitive dominance between resource species (Hanley & Sykes, 2009), potentially promoting coexistence, for example by favoring species that are poor competitors but which can cope with high enemy pressure (Kuang & Chesson, 2009).

Working at the same field site, Garzon‐Lopez et al. (2015) found that seed mortality caused by mammalian and insect seed predators in their three study species (all trees) was better explained by densities of both conspecifics and heterospecifics, rather than conspecific densities alone. If such effects are widespread in tropical forests they could act as an important structuring force. Since herbivores and other plant enemies are often specialized at the level of genus or family (e.g., Gilbert & Webb, 2007; Novotny et al., 2012), we would expect plant species to be linked to the density of (and distance to) closely related plant species, potentially leading to phylogenetic overdispersion of plant communities and ultimately increasing “phylodiversity” (Webb, Gilbert, Donoghue, & Ilbert, 2006). Uncovering the full role of indirect interactions will require additional experiments for a wider range of species, quantifying both the functional response of natural enemies to co‐occurring species, and the longer‐term consequences for plant population dynamics and diversity.

## CONFLICT OF INTEREST

None declared.

## AUTHOR CONTRIBUTIONS

HD, SG, OL, and MB conceived and designed the study. HD carried out data collection for all studies except study 2. CF carried out field work for Study 2 and undertook initial surveys of the study system. HD analyzed the data, interpreted the results, and drafted the manuscript with input from all authors.

## DATA ACCESSIBILITY

Data available from the Dryad Digital Repository: https://doi.org/10.5061/dryad.fd15ff0
