# Supplementary material for: Insect herbivory on seedlings of rainforest trees: Effects of density and distance of conspecific and heterospecific neighbors
Source: Ecol Evol. 2018 Dec 7;8(24):12702–11. doi: 10.1002/ece3.4698 (PMC6308876; doi:10.1002/ece3.4698)
Supplement: Supplementary file 1 [file ECE3-8-12702-s001.docx]

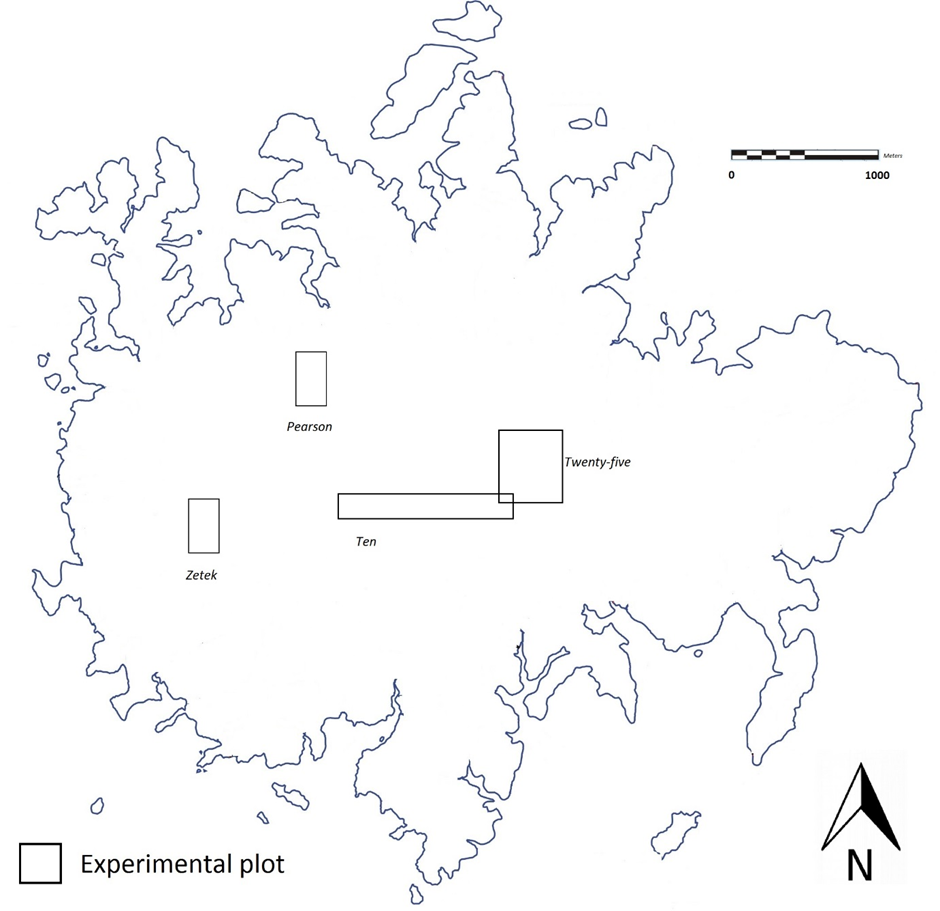


Supplementary Figure 1 - Map of Barro Colorado Island showing the location of experimental plots.
