# Supplementary material for: Insect herbivory on seedlings of rainforest trees: Effects of density and distance of conspecific and heterospecific neighbors
Source: Ecol Evol. 2018 Dec 7;8(24):12702–11. doi: 10.1002/ece3.4698 (PMC6308876; doi:10.1002/ece3.4698)
Supplement: Supplementary file 2 [file ECE3-8-12702-s002.docx]

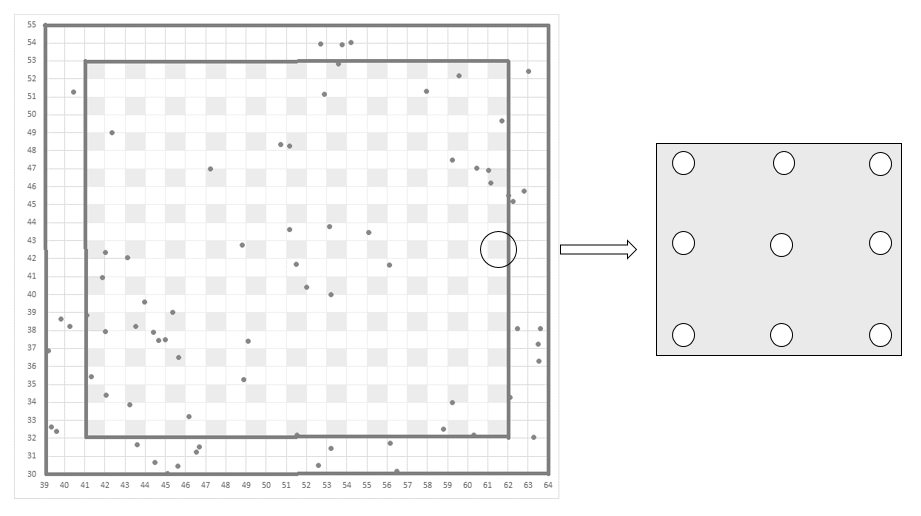


Supplementary Figure 2- The 20m × 20m grid system of the twenty-five hectare plot including the 40m buffer zone along the plot edges. Grey dots show the location of adult Cordia trees. Shaded squares indicate subplots where potted seedlings were introduced to nine locations according to the design on the right.
