# Supplementary material for: Insect herbivory on seedlings of rainforest trees: Effects of density and distance of conspecific and heterospecific neighbors
Source: Ecol Evol. 2018 Dec 7;8(24):12702–11. doi: 10.1002/ece3.4698 (PMC6308876; doi:10.1002/ece3.4698)
Supplement: Supplementary file 4 [file ECE3-8-12702-s004.docx]

Supplementary Table 1- Structure of the statistical models used, indicating the type of model used and the response and explanatory variables for both maximal and minimum models.

| **Study** | **Model Type** | **Error Structure** | **Response variable** | **Random effects** | **Fixed effects (maximal model)** | **Fixed effects (minimum adequate model)** |
| --- | --- | --- | --- | --- | --- | --- |
| Study 1 | GLMM | Quasibinomial (to account for underdispersion) | Seedling herbivory (proportion per subplot) | Plot number | Focal seedling treatment (high/low density) + summed basal area of *C. alliodora* adults in neighbourhood+ summed basal area of *C. bicolor* adults in neighbourhood. | Focal seedling treatment. |
| Study 2 | GLMM | Binomial | Seedling herbivory (proportion per subplot) | Plot number + to account for overdispersion in the data, an observation level random factor was included in the model. | Local density of *C. alliodora* seedlings (in 0.5m² subplot)+ local density of *C. bicolor* seedlings (in 0.5m² subplot) + summed basal area of *C. alliodora* adults (20m x 20m) and summed basal area of *C. bicolor* adults (in 20 x 20m plot). | Local density of *C. alliodora* seedlings (in 0.5m² subplot). |
| Study 3 | GLMM | Binomial | Herbivory (binary response) | Adult tree ID | Seedling distance (m) from adult tree +local seedling density + adult tree species (*C. alliodora*, *C. bicolor* or *G. standleyana*) + adult tree species*local seedling density | Adult tree species |
| Study 4 | GLM | Quasibinomial (to account for overdispersion) | Seedling herbivory (binary response) | NA | Location from adult tree (inside/outside seedling carpet) + focal seedling species (*C. alliodora* or *C. bicolor*)+ number of days focal seedling in field+ distance from adult tree* focal seedling species+ adult tree ID. | All terms, including the interaction term. |
| Study 4 | GLM | Binomial | Seedling survival (binary response) | NA | Herbivory by *I. annulus* (yes/no). | Herbivory by *I. annulus* (yes/no). |
| Study 5 | GLM | Binomial | Seedling survival (binary). | NA | Initial seedling state (intact/herbivory). | Initial seedling state (intact/herbivory) |
