# Supplementary material for: Insect herbivory on seedlings of rainforest trees: Effects of density and distance of conspecific and heterospecific neighbors
Source: Ecol Evol. 2018 Dec 7;8(24):12702–11. doi: 10.1002/ece3.4698 (PMC6308876; doi:10.1002/ece3.4698)
Supplement: Supplementary file 5 [file ECE3-8-12702-s005.docx]

Supplementary Table 2- Model outputs for spatial autocorrelation analysis

| **Model** |  | **Random Effects** | | **Fixed Effects** | | | |
| --- | --- | --- | --- | --- | --- | --- | --- |
|  |  | Variance | Std. deviation | Estimate | Std. Error | Z value | P value |
| Census Two | Intercept | 20.85 | 4.566 | -6.121 | 1.802 | -3.397 | 0.001 |
| Census Two | 10-20m | 20.85 | 4.566 | 1.728 | 1.458 | 1.186 | 0.236 |
| Census Two | 20-30m | 20.85 | 4.566 | -7.395 | 4.203 | -1.76 | 0.078 |
| Census Two | 30-40m | 20.85 | 4.566 | -5.339 | 6.4 | -0.834 | 0.404 |
| Census Two | 40-50m | 20.85 | 4.566 | 1.212 | 5.021 | 0.241 | 0.809 |
| Census Two | 50-60m | 20.85 | 4.566 | -22.5 | 10.921 | -2.06 | 0.039 |
| Census Two | 60-70m | 20.85 | 4.566 | 4.372 | 4.955 | 0.882 | 0.378 |
| Census Two | 70-80m | 20.85 | 4.566 | 2.309 | 5.867 | 0.394 | 0.694 |
| Census Two | 80-90m | 20.85 | 4.566 | -3.369 | 9.054 | -0.372 | 0.71 |
| Census Two | 90-100m | 20.85 | 4.566 | -48.23 | 20.011 | -2.41 | 0.016 |
| Census Three | Intercept | 0.5829 | 0.7635 | -3.14 | 0.562 | -5.586 | 2.32E-08 |
| Census Three | 10-20m | 0.5829 | 0.7635 | 1.192 | 0.566 | 2.108 | 0.035 |
| Census Three | 20-30m | 0.5829 | 0.7635 | 1.639 | 1.193 | 1.374 | 0.169 |
| Census Three | 30-40m | 0.5829 | 0.7635 | 3.356 | 1.191 | 2.819 | 0.005 |
| Census Three | 40-50m | 0.5829 | 0.7635 | -3.295 | 2.401 | -1.372 | 0.17 |
| Census Three | 50-60m | 0.5829 | 0.7635 | 1.914 | 2.059 | 0.93 | 0.353 |
| Census Three | 60-70m | 0.5829 | 0.7635 | 3.325 | 2.041 | 1.629 | 0.103 |
| Census Three | 70-80m | 0.5829 | 0.7635 | -1.507 | 2.694 | -0.559 | 0.576 |
| Census Three | 80-90m | 0.5829 | 0.7635 | -0.348 | 3.226 | -0.108 | 0.914 |
| Census Three | 90-100m | 0.5829 | 0.7635 | -4.357 | 2.904 | -1.501 | 0.133 |
| Census Four | Intercept | 0.3697 | 0.6081 | -2.3 | 0.552 | -4.161 | 3.17E-05 |
| Census Four | 10-20m | 0.3697 | 0.6081 | 0.279 | 0.482 | 0.058 | 0.954 |
| Census Four | 20-30m | 0.3697 | 0.6081 | 0.724 | 1.169 | 0.62 | 0.535 |
| Census Four | 30-40m | 0.3697 | 0.6081 | 0.841 | 0.943 | 0.892 | 0.373 |
| Census Four | 40-50m | 0.3697 | 0.6081 | 1.418 | 1.402 | 1.012 | 0.312 |
| Census Four | 50-60m | 0.3697 | 0.6081 | 0.826 | 1.492 | 0.553 | 0.58 |
| Census Four | 60-70m | 0.3697 | 0.6081 | 2.523 | 1.511 | 1.67 | 0.095 |
| Census Four | 70-80m | 0.3697 | 0.6081 | -1.842 | 1.888 | -0.976 | 0.329 |
| Census Four | 80-90m | 0.3697 | 0.6081 | 0.014 | 2.129 | 0.006 | 0.995 |
| Census Four | 90-100m | 0.3697 | 0.6081 | -2.01 | 1.781 | -1.128 | 0.259 |
| Census Five | Intercept | 0.1447 | 0.3803 | -1.574 | 0.401 | -3.929 | 8.54E-05 |
| Census Five | 10-20m | 0.1447 | 0.3803 | 0.054 | 0.421 | 0.129 | 0.898 |
| Census Five | 20-30m | 0.1447 | 0.3803 | 0.685 | 0.741 | 0.937 | 0.349 |
| Census Five | 30-40m | 0.1447 | 0.3803 | 1.42 | 0.783 | 1.814 | 0.07 |
| Census Five | 40-50m | 0.1447 | 0.3803 | 0.607 | 1.076 | 0.565 | 0.572 |
| Census Five | 50-60m | 0.1447 | 0.3803 | 0.263 | 1.115 | 0.235 | 0.814 |
| Census Five | 60-70m | 0.1447 | 0.3803 | 0.346 | 1.218 | 0.284 | 0.776 |
| Census Five | 70-80m | 0.1447 | 0.3803 | -0.95 | 1.309 | -0.726 | 0.468 |
| Census Five | 80-90m | 0.1447 | 0.3803 | -1.945 | 1.66 | -1.172 | 0.241 |
| Census Five | 90-100m | 0.1447 | 0.3803 | 0.146 | 1.357 | 0.107 | 0.914 |
| Census Six | Intercept | 0 | 0 | -1.319 | 0.38 | -3.472 | 0.001 |
| Census Six | 10-20m | 0 | 0 | 0.43 | 0.397 | 1.084 | 0.279 |
| Census Six | 20-30m | 0 | 0 | 1.907 | 0.616 | 3.095 | 0.002 |
| Census Six | 30-40m | 0 | 0 | 0.17 | 0.798 | 0.214 | 0.831 |
| Census Six | 40-50m | 0 | 0 | -1.803 | 1.1 | -1.64 | 0.101 |
| Census Six | 50-60m | 0 | 0 | -0.45 | 1.005 | -0.448 | 0.654 |
| Census Six | 60-70m | 0 | 0 | 1.278 | 1.095 | 1.167 | 0.243 |
| Census Six | 70-80m | 0 | 0 | 0.032 | 1.23 | 0.026 | 0.979 |
| Census Six | 80-90m | 0 | 0 | 0.726 | 1.439 | 0.504 | 0.614 |
| Census Six | 90-100m | 0 | 0 | -2.343 | 1.415 | -1.656 | 0.098 |
| Census Seven | Intercept | 0.2068 | 0.4548 | 0.48 | 0.639 | 0.752 | 0.452 |
| Census Seven | 10-20m | 0.2068 | 0.4548 | 0.214 | 0.338 | 0.634 | 0.526 |
| Census Seven | 20-30m | 0.2068 | 0.4548 | -1.073 | 0.606 | -1.771 | 0.077 |
| Census Seven | 30-40m | 0.2068 | 0.4548 | 0.142 | 0.6 | 0.236 | 0.814 |
| Census Seven | 40-50m | 0.2068 | 0.4548 | 1.103 | 0.813 | 1.356 | 0.175 |
| Census Seven | 50-60m | 0.2068 | 0.4548 | 0.013 | 0.869 | 0.015 | 0.988 |
| Census Seven | 60-70m | 0.2068 | 0.4548 | -0.52 | 0.938 | -0.555 | 0.579 |
| Census Seven | 70-80m | 0.2068 | 0.4548 | -1.411 | 1.088 | -1.297 | 0.195 |
| Census Seven | 80-90m | 0.2068 | 0.4548 | 0.938 | 1.328 | 0.706 | 0.48 |
| Census Seven | 90-100m | 0.2068 | 0.4548 | -1.019 | 1.102 | -0.924 | 0.355 |
