# Supplementary material for: Insect herbivory on seedlings of rainforest trees: Effects of density and distance of conspecific and heterospecific neighbors
Source: Ecol Evol. 2018 Dec 7;8(24):12702–11. doi: 10.1002/ece3.4698 (PMC6308876; doi:10.1002/ece3.4698)
Supplement: Supplementary file 6 [file ECE3-8-12702-s006.docx]

Supplementary Table 3 - Model outputs for the statistical analyses.

| Model | Final model | Response | Effects | Estimate | Std. Error | Z value |
| --- | --- | --- | --- | --- | --- | --- |
| Study 1 | Herbivory~ treatment | Herbivory | Intercept (high density)  Low density | -0.80  -0.43 | 0.15  0.21 | -5.18  -2.08 |
| Study 2 | Herbivory~ conspecific seedling density | Herbivory | Intercept  Number conspecific seedlings | -0.57  0.03 | 0.41  0.02 | -1.39  1.91 |
| Study 3 | Herbivory~ adult tree species | Herbivory | Intercept (*Cordia alliodora)*  *Cordia bicolor*  *Guapira standleyana* | 0.50  -7.56  -1.47 | 0.28  0.39  0.40 | 1.77  -1.92  -3.65 |
| Study 4 | Herbivory~ | Herbivory | Intercept (Species: *C. alliodora*, Distance: far, Site1)  Species (*C. bicolor)*  Distance (near)  Site 2  Site 3  Site 4  Number days  *C. bicolor* x near | -3.41  1.09  1.89  0.55  0.38  0.86  0.04  -1.09 | 0.48  0.12  0.13  0.12  0.12  0.13  0.01  0.18 | -7.04  9.14  14.97  4.53  3.15  6.89  4.64  -6.24 |
| Study 4 | Survival~ herbivory | Survival | Intercept  Herbivory | -4.23  4.55 | 0.26  0.27 | -16.28  17.18 |
| Study 5 | Survival~ herbivory | Survival | Intercept  Herbivory | -4.23  4.55 | 0.26  0.27 | -16.28  17.18 |
